# Advanced therapy screening in inflammatory bowel disease and the impact of clinical nurse specialists: A retrospective analysis of electronic patient records

**DOI:** 10.1016/j.clinme.2025.100317

**Published:** 2025-04-23

**Authors:** Michael Colwill, Arin Ward, Kevin Jacob, Richard Hall, Dara Rasasingam, Sarah O’Neill, Fiona Donovan, Jennifer Clough, Richard Pollok, Andrew Poullis

**Affiliations:** a City St George’s, University of London, Cranmer Terrace, London, SW17 0RE, UK; b Department of Gastroenterology, St George’s University Hospitals NHS Foundation Trust, Blackshaw Road, London, SW17 0QT, UK

**Keywords:** Inflammatory bowel disease, Clinical nurse specialist, Advanced therapy, Screening

## Abstract

Advanced therapies (AT), encompassing biologics and small molecules, are a common and important treatment for inflammatory bowel disease (IBD). However, these treatments pose a risk of reactivating latent infections and therefore require pre-treatment infection screening, but compliance with this screening has previously been reported to be poor. Clinical nurse specialists (CNS) and pharmacists play a key role in facilitating this screening and safely initiating AT, but are understaffed compared to national standards. Through retrospective review of electronic patient records at St George’s University Hospital, a tertiary IBD centre in London, UK, we evaluated the impact of staffing on rates of compliance with screening and time from prescription to administration of AT (TAT). 1,035 patients with IBD treated with an AT were identified, and we found a significant correlation between increased CNS staffing and improved screening compliance, as well as a numerical reduction in the TAT. Incidental findings were relatively low, with 8% of patients presenting positive results, all of whom had clinical risk factors. The study advocates for increased staffing and resources in IBD services to enhance patient safety and treatment efficacy.

## Highlights

• IBD clinical nurse specialists (CNS) play a pivotal role in starting advanced therapies in IBD, including the crucial step of pre-treatment infection screening to avoid reactivation of latent of infections.
• We have demonstrated that increasing staffing levels significantly improves compliance with pre-treatment screening.
• Improved staffing levels also reduce the time taken from the decision to start an advanced therapy to the initiation of treatment.
• Despite this, IBD services across the UK are understaffed with regards to CNS and specialist pharmacists.

## Graphical abstract

**Figure:**
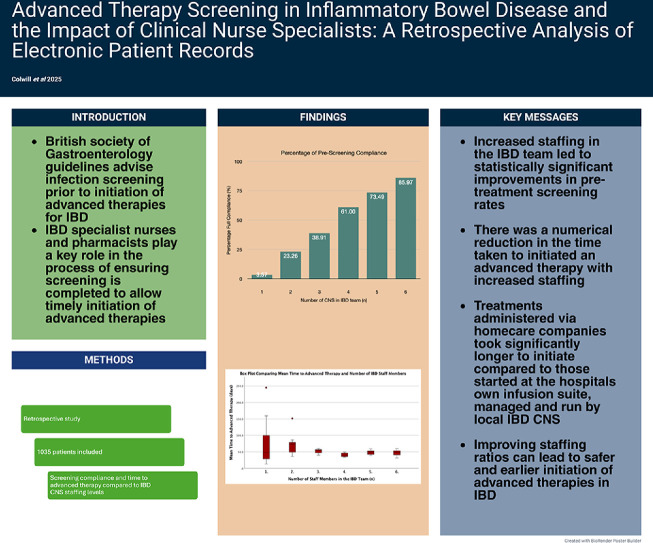

## Introduction

Inflammatory bowel disease (IBD) is a chronic inflammatory condition of the gastrointestinal tract encompassing ulcerative colitis and Crohn’s disease. Patients with a more severe disease phenotype are treated with advanced therapies (AT) comprising biologic medications such as infliximab, adalimumab, ustekinumab, risankizumab or vedolizumab, and small molecule treatments such as tofacitinib, filgotinib, upadacitinib or etrazimod.^1^ These medications inhibit different cellular targets in order to suppress immune-mediated inflammation within the gastrointestinal tract and alleviate symptoms.

Given this immunosuppressive effect, there is a risk of reactivation of previous infections with potentially life-changing consequences.^2^ The British Society of Gastroenterology (BSG) guidelines^3^ therefore advise that all patients being started on an AT should have a combination of clinical, serological and radiological screening tests to identify any evidence of infection with human immunodeficiency virus (HIV), hepatitis B virus (HBV), hepatitis C virus (HCV), varicella-zoster virus (VZV), herpes simplex virus (HSV) and tuberculosis (TB). This pre-treatment screening allows for treatment of these conditions prior to, or in parallel with, AT initiation, therefore reducing the risk to the patient of reactivation or disseminated infection.

However, due to a combination of both healthcare provider and patient factors, compliance with this screening is often poor, with data from the IBD registry in 2024 finding that only 59.4% of adult patients were fully screened prior to initiation of their AT.^4^ Given the potential serious adverse events that can occur from reactivation of these diseases, there is a need to increase AT screening rates and improve safety. There is also now a growing wealth of data suggesting that, for patients with more severe disease, earlier AT treatment is linked with better outcomes,^5^^,^^6^ but a failure to complete screening tests can result in delayed treatment initiation.

IBD clinical nurse specialists (CNS) play a key role at our institution in facilitating screening and initiating an AT. Once the clinical decision is made to initiate an AT by a physician, the patient is allocated to a designated IBD CNS or a specialist pharmacist, whose role is to ensure that all screening tests including a chest radiograph (CXR) have been requested, and to request them if required. They then contact the patient, explain the treatment and screening process and ensure that screening is completed. All screening tests including a CXR can be performed via a walk-in service at one of three hospital sites. The CNS or pharmacist will also review the test results and, if reported as normal, will then liaise with the patient to initiate therapy.

However, there are no published data on how IBD CNS and pharmacist staffing affects screening rates or the length of time from the decision to initiate an AT to administration of the first treatment, known as time to advanced therapy (TAT). We aimed to review the impact of staffing on both compliance with pre-treatment screening and TAT.

## Material and methods

### Study design

A retrospective analysis of electronic patient records, encompassing patient data from June 2011 to March 2024, was performed at St George’s University Hospital in London, UK, a tertiary IBD referral centre.

### Patient population

All patients over the age of 18 with confirmed IBD under the care of the gastroenterology team at St George’s Hospital as of March 2024 and currently being treated with an AT were included. Patients who had their index AT either started at another institution, as part of a clinical trial or as an inpatient were excluded.

### Data collection

Data were collected on demographic information, including the type of IBD, age at time of index AT initiation, sex and ethnicity, and the results from screening tests. Screening tests were defined as serology for HBV (both surface antigen and core antibody), HCV, HIV, VZV, interferon-gamma release assay (IGRA) for TB and a CXR. Full compliance with BSG guidance was defined as having been tested for all of HBV, HCV, HIV, VZV, IGRA and having had a CXR performed; partial compliance was if some, but not all, of these tests had been performed. The TAT for this index AT was also recorded, defined as time from clinical decision to initiate the AT to the date of their first infusion, injection or tablet. For the TAT analysis, patients with a positive screening test were excluded in addition to the above exclusion criteria. IBD CNS and pharmacists were not responsible for writing the prescriptions during the time period of our study.

Data were collected on current and historic levels of staffing within the IBD team. Incidental findings were defined as any positive result for HBV, HCV, HIV or TB which was not previously known, as well as ‘VZV status’ with previous exposure demonstrated through a positive IgG titre. For all positive results, patient electronic notes were reviewed to identify risk factors for these diseases.^7^, ^8^, ^9^

### Statistical analysis

Statistical analysis was performed using IBM SPSS Statistics version 29 for Mac. Descriptive statistics were provided in the form of mean compliance rates and median TAT per calendar quarter. Rates of compliance were compiled for each calendar quarter and compared to the number of CNS in the IBD team. The Kruskal–Wallis test was used to compare the differences in compliance rates for different levels of staffing and 95% confidence intervals were provided. Statistical significance was defined as *p*≤0.05.

## Results

### Baseline characteristics

1,298 patients were identified as currently being treated with an AT for IBD. 263 of these patients were excluded due to their index AT being started at another healthcare facility, available data being incomplete, or being treated in a clinical trial. 1,035 were therefore included in the analysis and baseline demographics are summarised in table 1.

**Table 1:** Demographic details of the studied population.

Demographics	
**Disease type**	**n (%)**
Ulcerative colitis	368 (35.6)
Crohn’s disease	657 (63.5)
IBD-U	10 (0.9)
**Sex**	**n (%)**
Male	552 (53.3)
Female	483 (46.7)
**Age at time of AT initiation**	
Minimum	18
Maximum	82
**Ethnicity**	**n (%)**
White	725 (70.0)
Black	79 (7.6)
Asian	197 (19.0)
Mixed	34 (3.3)
**Treatment**	**n (%)**
Infliximab	331 (32.0)
Adalimumab	394 (38.1)
Ustekinumab	130 (12.6)
Vedolizumab	49 (4.7)
Tofacitinib	75 (7.3)
Filgotinib	45 (4.4)
Risankizumab	10 (1.0)
Upadacitinib	1 (0.1)

The first patient was initiated on an AT in 2011. The median age at initiation was 36.2 years.

### Staffing levels and screening completion

The staffing levels of the IBD team are summarised in Table 2. The sixth member of staff added in Q4 of 2022 was a dedicated IBD pharmacist who performed the same roles as a CNS and did not prescribe at the time of data collection, and their data have therefore been included in the analysis alongside CNS data.

**Table 2:** Screening completion rates and TAT compared to staffing within the IBD team over time.

**Staff in IBD team (n)**	**Dates**	**Months**	**Patients started on AT (n)**	**Patients started on AT per month (n)**	**Patients with complete screening (%)**	**Patients with partial screening (%)**	**Patients with no screening (%)**	**Mean TAT (Days)**
**1**	2011 Q1 – 2014 Q4	48	38	0.8	7.9	71.1	21.1	71.3
**2**	2015 Q1 – 2018 Q3	45	209	4.6	32.5	63.2	4.8	70.3
**3**	2018 Q4 – 2019 Q4	15	157	10.5	38.9	61.2	0	51.8
**4**	2020 Q1 – 2020 Q4	12	156	13	62.2	37.2	0.6	40.6
**5**	2021 Q1 – 2022 Q3	21	275	13.1	77.8	20.7	1.5	48.3
**6**	2022 Q4 - 2024 Q2	18	200	11.1	88.5	11.4.	0.1	47.0

IBD, inflammatory bowel disease; TAT, time to advanced therapy.

The rates of full screening improved with the addition of each new staff member, while the rates of partial and absent screening reduced. These results are summarised in Table 2.

**Fig. 1:**
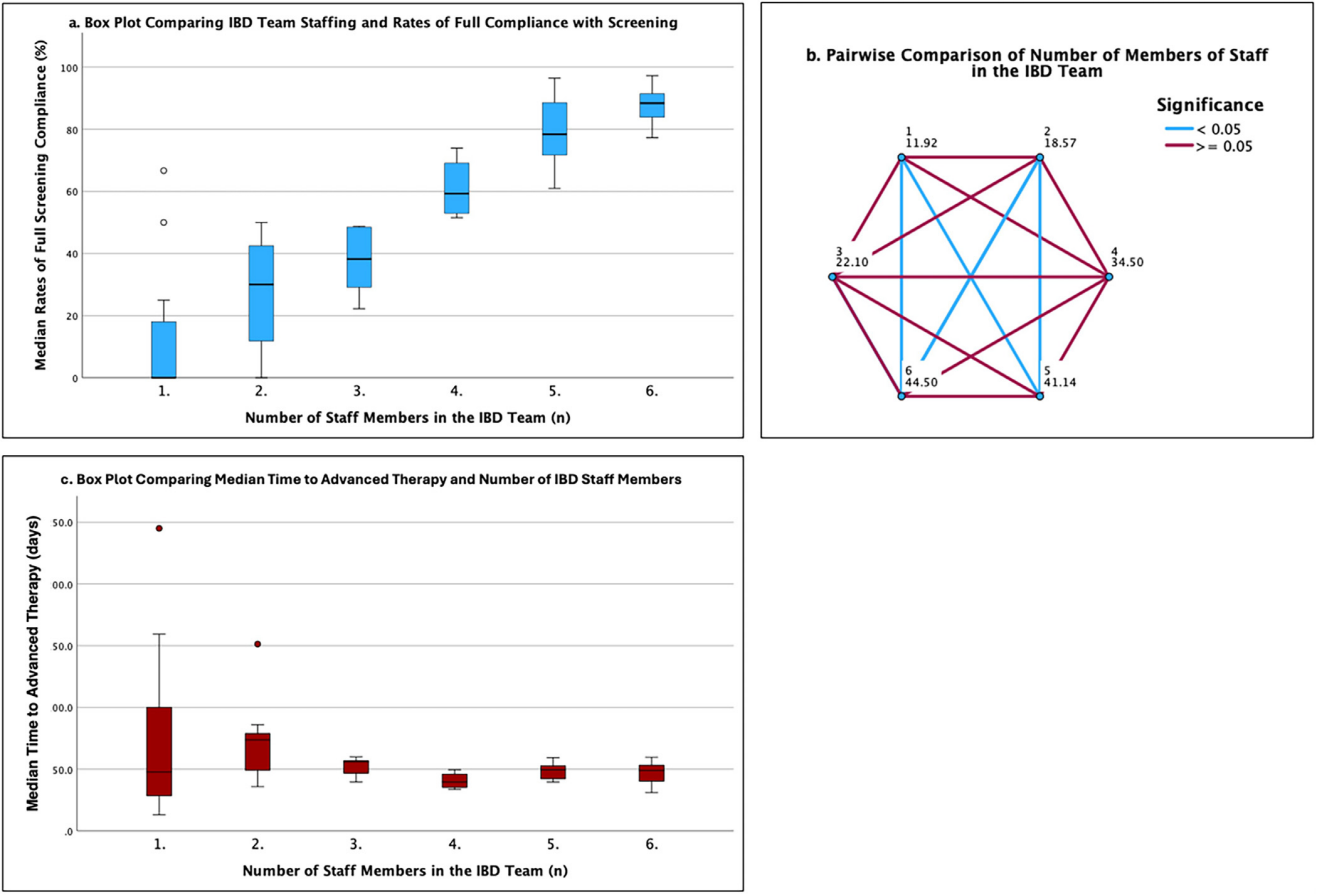
(a) Box plot comparing IBD team staffing with rates of full compliance with screening. The horizontal black lines denote median values; boxes extend from the 25th to the 75th percentile of each group’s distribution of values; vertical extending lines denote minimum and maximum values that are not outliers, dots denote outliers. (b) Pairwise comparison of number of members of staff in the IBD team with regards to improvement in compliance with screening. (c) Box plot comparing IBD team staffing with rates of time to advanced therapy. The horizontal black lines denote median values; boxes extend from the 25th to the 75th percentile of each group’s distribution of values; vertical extending lines denote minimum and maximum values that are not outliers, dots denote outliers.

A Kruskal–Wallis test showed that increases in staff numbers were associated with a statistically significant (*p*≤0.01) increase in full compliance with screening (Fig. 1a and b).

Data were not available on why patients did not comply with their screening tests, or on which specific tests were not completed for partially completed screening.

### Staffing levels and average time to AT

952 patients were included in the TAT analysis. The median TAT for all patients was 35 days (range 5–313 days; interquartile range (IQR) 42). An increased number of staff members was associated with a numerical decrease in TAT, with the exception when increasing from four staff members to five. A Kruskal–Wallis test showed that these results were not statistically significant (*p*=0.188) (Fig. 1c). The results are summarised in Table 2.

Patients who were treated with an AT administered by homecare companies (adalimumab, tofacitinib, upadacitinib and filgotinib) had a median TAT of 42 days (range 5–313; IQR 45). This was significantly longer than those who had treatment initiated at the hospital infusion suite (infliximab, risankizumab, ustekinumab, vedolizumab) which was 30 days (range 5–255; IQR 30.25; *p*=0.000002). Sub-group analysis of the homecare-administered AT also showed that the median TAT for adalimumab, which is administered subcutaneously, was 48 days (range 5–313; IQR 44.5) which was significantly longer than the those treated with oral therapies, where the median TAT was 26 days (range 5–262; IQR 33; *p*=0.0000039).

### Incidental findings

The total number of incidental findings from screening tests was 83 (8.02%). The individual breakdowns are listed in Table 3. 193 (18.6%) patients tested negative for VZV. Of the 26 abnormal CXR, two identified incidental findings of malignancy – one breast cancer and one lung cancer.

**Table 3:** Breakdown of incidental findings from advanced therapy screening.

Test	Number of positive results n (%)
**HBsAg**	8 (0.8)
**HBcAb**	7 (0.7)
**Hepatitis C antibody**	1 (0.1)
**HIV antibody**	2 (0.2)
**IGRA**	39 (3.8)
**Chest radiograph**	26 (2.5)
**VZV IgG**	842 (81.4)
**Total**	83 (8.0)

## Discussion

Our study using real-world data over a period of 13 years has demonstrated, to our knowledge for the first time, that increased staffing is associated with improved screening completion rates and a numerical reduction in the TAT, despite the number of AT prescriptions per month increasing. Given their role in liaising directly with patients, answering questions and chasing test results, we believe that our findings present empirical data of the value of CNS and pharmacists in providing high-quality care to patients with IBD. The incidental pick-up rate from screening was relatively low at 8.0% and a total of 18.6% of patients lacked serological evidence of previous VZV exposure.

The IBD-UK standards suggest that there should be 2.5 full-time IBD CNS per 250,000 people in their local population.^10^ For our service, this would be 13 full-time IBD CNS and our staffing falls short of this recommendation. This is a common theme nationally, with only 14% of IBD services meeting this threshold. Our data demonstrate that improving staffing closer to the IBD-UK standards can improve care.

There is evidence that increased IBD team staffing can improve the quality of care provided by IBD services,^11^^,^^12^ but the impact on the TAT has not previously been assessed. While there was a numerical trend showing that increased IBD team staffing reduces TAT, we did not find a statistical correlation. This may be because the processes that require initiation of an AT are not solely the responsibility of the IBD team and therefore external delays, which are not affected by IBD team staffing levels, continue to drive delay. An example is the supply, training and administration of adalimumab, the first-line AT at our institution for Crohn’s disease, which is managed by an external homecare company which initiates the process once they have received authorisation from the medical team. The same provision process is utilised for small molecule treatments such as JAK inhibitors, whose use has significantly increased at our institution for the past 2 years. Therefore, even if the consent, screening and administrative paperwork is performed more quickly, this outsourcing process is likely still incurring independent delays.

This outsourcing process has recently been the subject of House of Lords committee review, which advised that the current homecare system introduces delays, complexity and a lack of accountability.^13^ These challenges will likely only continue to grow as more patients are started on oral therapies or receive subcutaneous, rather than intravenous, induction therapy at home via a homecare company. Our data are in keeping with the concerns highlighted in this report regarding delays due to the outsourcing process, as we have demonstrated a significantly longer TAT for AT administered via homecare therapies compared to those administered at our hospital infusion suite. It is also important to note that at our institution the infusion suite is managed and run by IBD CNS, and the quicker initiation of AT in this service is further evidence of the value of improved CNS staffing to patient care.

Our study also found a relatively low pick-up rate of incidental infections. 3.8% of patients tested positive for latent TB and this is slightly higher than identified in previous work from the Netherlands, which found a prevalence of 3%,^14^ but probably reflects the higher risk population served in south-west London.^15^ The prevalences of HBV (1.5%) and HCV (0.1%) are lower than in previous studies,^16^ which may be due to the advent of direct-acting antivirals and more proactive screening programmes, particularly in London.^17^^,^^18^ The rate of incidental HIV infection was 0.2%, which is lower than the population-wide rates of infection seen across London.^19^ The reasons for this are unclear, but may be due to enhanced screening programmes in London, such as testing all emergency department attendees. Upon reviewing the electronic patient records for these patients, all were found to have clinical risk factors for infection as previously described.

Given that clinical trial data show significant levels of VZV reactivation with JAK inhibitors and potentially significant harm to patients,^20^ absence of previous exposure to VZV necessitates vaccination prior to initiation. While national data suggest that approximately 90% of adults in the UK are seropositive for the VZV IgG,^21^^,^^22^ our data show a lower prevalence at 81.4%. The reasons for this are unclear, but may be related to the diverse nature of our local population, with previous work having shown that seropositivity is lower in non-white individuals.^23^

At our institution, it can often takes several days for screening results to be returned and in some circumstances, such as acute severe ulcerative colitis, this delay can be severely detrimental to the patient’s outcome and result in the need for a colectomy. Given the relatively low prevalence of latent infection and the universal presence of risk factors in those with underlying latent infection, our data suggest that, for patients requiring urgent administration of an AT, initiation of treatment could safely proceed prior to the results of screening tests being available with careful clinical assessment and case selection.

The strengths of our study are the large number of participants and the long period of data inclusion. We have also compared characteristics that have not been studied previously, and have demonstrated with an evidence base the positive impact that improved staffing can have on the care provided to patients with IBD.

While a large dataset, it is limited to a single centre in an urban environment and the findings may not be generalisable to the wider UK population, particularly with regards to the incidental findings rate. Our data cover a time period of over 13 years, during which time clinical practice has significantly changed. Physicians are more familiar with using anti-TNF treatments and processes to expedite the first administration, such as screening and infusion suite appointment booking, are now better understood and utilised. Recent research has also increased awareness among IBD healthcare providers with regards to the urgency of starting AT because of the benefits of earlier treatment^5^ and risks of delayed treatment.^6^ While we know that these factors may have impacted upon our results with regards to both screening compliance and TAT, we are not able to account for this. There were also periods where some staff members were on extended leave and this was not factored into our analysis. We did not collect data on rates of infection reactivation among our entire IBD patient population, so we cannot demonstrate a change in adverse events associated with improvements in screening.

Further study of the effect of CNS staffing on rates of reactivation of latent infections, as well as data from other centres across the UK, is required to further demonstrate the positive impact that sufficient IBD CNS staffing has for patient care.

## Conclusion

Increased staffing among the IBD team is associated with improved rates of screening and a shorter TAT. We advocate for sufficient funding for IBD services to facilitate adequate staffing provision and to help ensure safe and timely treatment for patients requiring an AT.

## Funding

This research did not receive any specific grant from funding agencies in the public, commercial or not-for-profit sectors.

## Ethics approval and consent to participate

The study was reviewed by the St George’s, University of London Research Ethics Committee (reference SE0125) who felt that, given the type of the study and anonymised data, ethical approval and patient consent were not required for this study.

## CRediT authorship contribution statement

**Michael Colwill:** Writing – review & editing, Writing – original draft, Project administration, Investigation, Formal analysis, Data curation. **Arin Ward:** Writing – review & editing, Investigation. **Kevin Jacob:** Writing – review & editing, Investigation. **Richard Hall:** Writing – review & editing, Investigation. **Dara Rasasingam:** Writing – review & editing, Investigation. **Sarah O’Neill:** Writing – review & editing, Investigation. **Fiona Donovan:** Writing – review & editing, Investigation. **Jennifer Clough:** Writing – review & editing. **Richard Pollok:** Writing – review & editing. **Andrew Poullis:** Writing – review & editing, Supervision, Conceptualization.

## Declaration of competing interest

The authors declare that they have no known competing financial interests or personal relationships that could have appeared to influence the work reported in this paper.
